# Prevention of the Choking Game: parent perspectives

**DOI:** 10.5249/jivr.v4i2.119

**Published:** 2012-07

**Authors:** Jessica M. Bernacki, W.Hobart Davies

**Affiliations:** ^*a*^University of Wisconsin-Milwaukee, Wisconsin, USA.

## Abstract

**Background:**

Many preadolescents and adolescents have been reported to take part in forced asphyxiation as a means of creating a feeling of being high without taking drugs. This activity goes by different names, including the Choking Game, Blackout, and Space Monkey. The limited epidemiological data suggest that about 6-11% of adolescents report having engaged in this behavior.

**Methods:**

This study surveyed a predominantly Caucasian cohort of parents regarding their knowledge of the choking game and its associated risks, as well as their attitudes toward possible prevention efforts.

**Results:**

Three quarters of parents responding reported being familiar with the choking game but considerably fewer (20%) reported having talked to their children about this activity. Ninety-six percent of parents reported knowing that unintentional death was a potential risk and ninety percent believe information about this activity should be included in school health curricula.

**Conclusion:**

Parents of adolescents in the United States appear to be quite knowledgeable about the Choking Game and its potential risks and are overwhelmingly supportive of prevention measures. The parents surveyed understood the importance of preventing children from engaging in the Choking Game, but may need specific help in how to talk to their children about it. Further work is needed to confirm that the proportion of parents identified as aware of this risk taking behavior is consistent across other populations and to urgently identify effective prevention efforts that can be integrated into existing health curricula.

## Introduction

The Choking Game is a thrill-seeking behavior in which forced asphyxiation is used as a means of creating the sensation of being high without taking drugs. This feeling is achieved by applying pressure to the neck with the use of hands or ligatures that restrict oxygen flow to the brain, or by putting pressure on the chest after hyperventilating. Participants’ describe experiencing a brief feeling of euphoria before they lose consciousness and again when the blood surges back to the brain when consciousness is regained. This activity goes by many different names, including the Choking Game, Blackout, the Fainting Game, and Space Monkey. When referring to this type of activity, Katz and Toblin have encouraged the use of the term “strangulation activity” rather than the “choking game” to convey the dangerousness of this behavior.^1^Since this paper addresses community awareness of this activity, the term the Choking Game will be used throughout this manuscript.

Limited epidemiological data exists for this activity and the empirical studies that have been completed have occurred in only the United States and Canada. A conservative estimate of 82 deaths between 1995 and 2007 has been suggested by the Center for Disease Control and Prevention (CDC), although advocacy groups estimate that rates exceed 100 each year with numerous cases misclassified as suicides (www.stop-the-choking-game.com). Such advocacy websites also include information about reported victims of the Choking Game, occurring as early as 1934 and in 25 countries. In 2006, a survey of adolescents completed in Ohio found that 11% of youth reported participating in the Choking Game.^2^A school-based study completed in Texas, and Ontario, Canada surveyed children between the ages of 9 to 18 years and found that 7% of these youth reported having engaged in the Choking Game, 45% reported knowing someone else who had, and 68% had heard of such activities.^3^In 2008, the Oregon Healthy Teens survey, completed by 8th graders, included a question about the Choking Game.^4^ In this statewide representative sample, 6% of youth had participated, 30% knew of someone participating, and 36% had heard of this activity. From this research, awareness of these activities appears to be common among adolescents, but there is a paucity of research about awareness and knowledge of risks in adults and youth of other ages.

The Choking Game seems to begin in groups, with some individuals later engaging in this behavior alone, which significantly raises the risk of unintentional death or disability. Recently, an increase in deaths associated with solo participation has been reported, but this may be in part due to better classification of cases previously misidentified as suicides.^5^Several case reports have been published that describe unintentional deaths resulting from engagement in this activity while alone.^6-8^Videos of the Choking Game are also widely available on the internet and demonstrate various methods for engaging in this behavior both in groups and while alone.^9^

Risks of this activity can include bruises, short term memory loss, seizures, concussions, retinal hemorrhage, stroke, brain damage, and brain death.^10^ A summary of the current understanding of the risks and signs of this behavior in older children and adolescents is available by Andrew, Macnab, and Russell.^11^Warning signs of solo participation may include: bruising or red marks around the neck, presence of items that appear to have been used as a ligature (e.g. belts, rope, ties, and clothing) disorientation after being alone, behavior changes, and bloodshot eyes.^11,12^

There is no current literature about parents' awareness of the Choking Game or attitudes about prevention. The goal of the current study is to describe parents’ awareness and views about prevention of this activity in a large community sample. This information will add to the current literature by providing the first examination of parents’ awareness and knowledge of potential risks, which can be used to inform future prevention efforts. Recent research has shown that physicians are aware of the need to screen for participation in these types of asphyxial activities during routine medical appointments, but no current research has examined such awareness in a parent population.^13^

## Methods

**Participants**

Participants were 1227 parents with children between the ages of 2 and 17 years (M = 9.34, SD = 5.44). Response rate was not tracked for this particular study, but in another study using this methodology, the response rate was 54%. Participants were between the ages of 18 and 62 years (M = 38.24, SD = 9.13) and their average years of education was 15.10 (SD = 2.31 years). The majority were mothers (64%), with 781 mothers and 442 fathers participating. Four participants did not report their gender. Participants were predominantly Caucasian (87%), but also included individuals who identified as African-American (7%), Hispanic/Latino (4%), Asian (2%), and individuals reporting as Multiracial or Other (4%). Parents had an average of 2.67 children (SD = 1.26) with a range of 1 to 7. Parents of children between the ages of 2 to 17 years were included to allow for a more generalizable assessment of awareness in the community. Participants were predominantly from Wisconsin (82%), with other participants living in other Midwest states (10%), outside of the Midwest but in the United States (8%), or outside of the United States (0.2%).Participants were recruited by students taking part in an advanced psychology laboratory class between 2008 and 2010. Each student enrolled in the class was required to recruit up to eight participants to complete the on-line survey.

**Procedure**

The project was approved by the Institutional Review Board and all students collecting data received training in the ethical conduct of research. Students in the class approached potential participants and asked them to take part anonymously in the study. They were given an information sheet that outlined the required elements of informed consent. Inclusion criteria included being in the targeted age range and being English speaking. After receiving verbal consent, participants were given instructions for accessing the survey at SurveyMonkey.com. On the first page of the survey, participants documented consent by confirming that they were over 18 and completing the survey voluntarily. Participants without internet access or who preferred not to participate online for other reasons were given the option to complete the forms on paper.

Descriptive questions about participants’ awareness and previous experience of the Choking Game were developed for this study. Information about potential risks associated with participation was taken from the Games Adolescents Shouldn’t Play (GASP) website (www.stop-the-choking-game.com). It is important to remember that these complications are based on anecdotal reports and the actual incidence of these complications has yet to be examined epidemiologically.

**Analyses**

Data analyses included summary statistics, frequencies, and proportions for categorical data which describes participants’ previous experience with the choking game, their perceptions of prevention efforts, and awareness of potential risks. Phi analyses were used to examine differences in mothers’ and fathers’ responses.

## Results

**Awareness**

Three-quarters of parents reported knowing of the Choking Game, and mothers were more likely to be aware of this activity (79%) than fathers (66%; Φ = .140, p<.01). When parents were asked if they had discussed this activity with their children, one-fifth of parents responded affirmatively, with mothers (24%) being more likely to have had this conversation than fathers (15%; Φ = .109, p< .01). Parents were also asked about their familiarity with the Choking Game during their own childhood. Twenty-seven percent of parents reported that they had been familiar with these types of activities as youth, 8% reported having participated directly while a child, and 1.4% reported having ever participated alone. A greater number of parents knew someone else who had participated in this activity (19%) and reported having heard of someone who may have died participating in this activity (15%). Fewer parents reported knowing someone personally who had died (4%) or knowing someone who sustained permanent disability from participating (1%). Responses to all questions divided by mother versus father report are included in Table 1.

**Table:** Table 1: **Survey results of parent’s knowledge and awareness of the Choking Game **

Question	Mothers	Fathers	phi
When growing up, heard of the Choking Game	25%	29%	-.05
When growing up, participated in the Choking Game	7%	11%	-.07
Ever participated in the Choking Game	7%	10%	-.05
Ever participated alone in the Choking Game	1%	2%	-.04
Know someone who participated	18%	22%	-.05
Know someone who participated alone	4%	3%	.02
Know someone disabled by participating	1%	1%	-.01
Knew of someone who died	16%	15%	.01
Knew someone personally who died	4%	3%	.04
Talked with their children about	24%	15%	.11^**^

Note. *p <.05, ** p<.01

**Perceptions of Risks**

After reading a description of the Choking Game (see the Appendix), parents were given a list of risks and asked to report which they perceived as possible consequences of this behavior. Ninety-six percent of parents perceived unintentional death was a potential risk, 95% loss of brain cells, 91% short term memory loss, 88% mental disability, 85% decreased academic potential, 82% seizures, and 78% physical disability. 

**Prevention Efforts**

Parents were also asked to provide their opinions about three proposed prevention efforts. Parents were overwhelmingly supportive of including education about the Choking Game into drug prevention programming (e.g., DARE) and school health curricula (see Table 2). A majority of parents thought that these education interventions should occur during middle school/junior high (62%), although 28% thought education should occur even earlier, during elementary school. Fewer parents reported that this education should wait until high school (4%), or that information about the Choking Game should not be included at all (5%). Parents were also asked if they thought that websites promoting these activities or displaying videos of participation should be banned from the internet. Parents were again overwhelming supportive of this prevention measure (87%). Mothers and fathers were equally supportive of the inclusion of the Choking Game into the health curriculum, but mothers were significantly more likely to be in favor of its inclusion in drug education programming (Φ=.106, p<.01) and for blocking websites promoting or showing the Choking Game (Φ=.110, p < .01). Parents who had participated in the Choking Game themselves when they were young, were significantly less likely to support the inclusion of such activities in health curricula compared to other parents (78% and 91% respectively; Φ =-.121, p < .05).

**Table:** Table 2:**Survey results of parents’ perceptions of prevention efforts**

Question	Mothers	Fathers	phi
Information about the Choking Game should be included in drug education programs	90%	82%	.11^**^
Information about the Choking Game should be included in school health curricula	91%	89%	.05
Sites should be banned that include information about the Choking Game	90%	82%	.11^**^

Note. *p <.05, ** p<.01

## Discussion

This study provides the first report of United States parents’ awareness of the Choking Game. Unintentional injuries are the leading cause of death and disability in children and adolescents with approximately 12,000 youth between the ages of 0 to 19 years dying each year.^14^Among adolescents, thrill-seeking and risk-taking behaviors in particular are associated with incidences of morbidity and death.^15^ A majority of the parents in this sample were familiar with the Choking Game, with a minority having participated themselves.

Similar to the research completed with physicians, few parents reported discussing the Choking Game with their children even though a majority reported being aware of this activity.^13^ Parents were overwhelmingly supportive though of implementing prevention efforts for such activities including drug education programming and inclusion in health curricula. Parents who had participated in the Choking Game as children were less likely to support their inclusion in health curricula than parents without childhood experience, although 75% still supported this prevention effort. Parents were also overwhelmingly supportive of banning “how to” videos on the internet, which although clearly desirable, is an action that is difficult to control.^9^These findings are consistent with previous reports of parents’ perceptions of drug abuse education in which they perceived such programs as providing their children with greater understanding of the risks of substance use and improved ability to resist participation.^16^ It is encouraging though, that even those parents who were originally unfamiliar with the Choking Game clearly recognized the need for prevention efforts once they learned more about this risk-taking behavior. Increasing parents’ awareness of such activities can provide them with the information they require to adequately monitor for signs of such risk-taking behaviors and prevent potentially life-threatening consequences. The Wisconsin Prevent Violence Against Children Act (2005) provides a strong model for prevention programming.^17^This Act required that the state of Wisconsin implement educational programs to inform people about the dangers of Shaken Baby Syndrome (SBS). These programs include: requiring all parents of newborns be provided educational information about SBS within seven days of their child’s birth; all day care providers must undergo specialized child safety training; and all students will receive education about SBS as part of both their middle school and high school coursework. Similar legislation for the Choking Game would allow for parents, youth, and medical care providers to obtain a greater understanding of the warning signs and risks of such activities and quickly spread awareness about this potentially lethal risk-taking behavior.

**Limitations**

Several limitations of the current study should be noted. The sample was predominantly Caucasian and well-educated, so conclusions regarding the apparent broad awareness of this activity should be cautiously applied to other racial, ethnic, and educational backgrounds. In general, the current literature regarding the Choking Game is limited across many demographic domains, so future research should explore if rates of participation, awareness, and incidence of morbidities and mortalities may show greater variability in more diverse samples. Unintentional injury in children is an important international health topic^18,19^and risk behaviors like the Choking Game should be examined cross-culturally. It would also be beneficial to collect information from parents who have children who are in the age range at risk for this behavior. Another limitation of the current study is the leading nature of the survey description (see Appendix). In our introduction to the Choking Game survey, participants were given a definition of this activity, including some potential risks, which could have increased participants' rate of reported risk awareness. Also, due to the recruitment approach utilized there may be a selective bias in who was willing and able to complete an online questionnaire. 

**Future Directions**

This study adds to the current literature by being the first examination of United States parents’ awareness of the Choking Game. Together with physicians, parents are in a central position to provide educational information to youth about the consequences of such risk activities, and provide the monitoring necessary to detect warning signs of participation.^11,13^ Parental supervision is particularly important for youth who may begin to engage in this activity alone, which significantly increases their risk of death or disability. This discrepancy between awareness and providing guidance is clinically important and future research should focus on addressing how parents can provide both this supervision and education.

## Appendix

**Survey Description of the Choking Game**

This section of the survey asks you some questions about The Choking Game described in the last section. This is known by a variety of names such as the Choking Game, the Fainting Game, Passout, and Space Monkey. The suffocation is usually accomplished either by strangling themselves with a belt or other object, or by having others push on their chest after hyperventilating. This is usually done in a group setting initially, but many people begin to do it by themselves. It is believed that some people get addicted to the "high" or lightheaded feeling and have difficulty stopping the behavior. Contrary to the belief that this is a "safe" alternative to drugs, this is actually quite dangerous. Even when it goes as planned, the Choking Game causes damage to the brain, and this adds up over time. Many youth experience seizures as they are waking up. Especially when done alone, the risk of death by strangulation is significant. While hundreds of deaths from this activity have been identified around the world, it is thought that many other cases are misidentified as suicides.
